# Clinicopathological analysis of 67 cases of esophageal neuroendocrine carcinoma and the effect of postoperative adjuvant therapy on prognosis

**DOI:** 10.1097/MD.0000000000027302

**Published:** 2021-10-29

**Authors:** Shenxiang Liu, Xiaolin Ge, Zhenzhen Gao, Qing Zhou, Yu Shi, Wangrong Jiang, Min Yang, Xinchen Sun

**Affiliations:** a Department of Radiotherapy, The First Affiliated Hospital of Nanjing Medical University, Jiangsu, China; b Department of Oncology, The Affiliated Hospital of Yangzhou University, Yangzhou University, Jiangsu, China; c Department of Oncology, The Second Affiliated Hospital of Jiaxing University, Zhe Jiang, China; d Department of Oncology, Jiangsu Provincial Tumor Hospital, Jiangsu, China; e Department of Radiotherapy, People's Liberation Army (PLA) 81 Hospital, Jiangsu, China; f Jiangsu Institute of Nuclear Medicine, Jiangsu, China.

**Keywords:** adjuvant therapy, esophageal, neuroendocrine carcinoma (NEC), prognosis, surgery

## Abstract

The clinicopathological properties of esophageal neuroendocrine carcinoma (ENEC) and its optimal therapy have not been widely studied, as the disease is not common. Consequently, we conducted a retrospective study to analyze the clinical features as well as the prognosis of patients with surgically resected ENEC.

The clinicopathological data of patients with ENEC who underwent esophagostomy with regional lymphadenectomy at Jiangsu Province People's Hospital and Jiangsu Provincial Tumor Hospital starting January 2008 until December 2014 were collected.

Ninety-two cases of ENEC were part of this study. However, only 67 patients were analyzed and followed up. A univariate model for the Cox proportional hazards revealed that prognosis was associated with postoperative adjuvant therapy, age, and lymph node metastasis (*P* < .05); a multivariate Cox proportional hazards model showed that postoperative adjuvant therapy was a significant independent prognostic factor. Postoperative adjuvant therapy directly affected overall survival, with a significant disparity noted between the groups (*P* = .022). In this study, patients who received adjuvant therapy had an average time of survival of 39 months (interquartile range: 27.068–50.932 months), while those who did not receive adjuvant therapy had an average survival time of 13 months (interquartile range: 10.129–15.871 months). The survival time was longer in the treated group than in the untreated group (hazard ratio = 0.47; 95% confidence interval: 0.23–0.94; *P* = .034).

ENEC is a heterogeneous tumor with a very poor prognosis. Combining surgery with adjuvant and/or chemotherapy significantly prolongs the survival of patients, and the optimal treatment for ENEC should be determined with future prospective studies.

## 1 Introduction

Neuroendocrine carcinoma (NEC) is a class of precursor tumors that use amines to synthesize and secrete heterogeneous amine and peptide hormones through decarboxylation. Mckeown^[1]^ first reported 1 cases of esophageal NEC (ENEC) in 1952; since then, it has been reported worldwide. According to the classification of digestive tract neuroendocrine tumors in 2010, NEC is a type of G3 neuroendocrine neoplasm (20 mitotic figures/10 high-power fields, Ki-67 positive index >20%), including small cell NEC, large cell NEC, and hybrid NEC.^[2]^

The incidence of ENEC accounts for approximately 2.5% to 5.9% of all esophageal cancers (ECs)^[3]^ and has been increasing over time.^[4,5]^

Because of the rare occurrence of this neoplasm, it lacks detailed clinicopathological features, prognostic data, and treatment strategies.^[6]^ To explain the clinicopathological features of ENEC, as well as the optimal treatment methods, there is a great need for more clinical samples of ENEC. Therefore, a retrospective analysis of the clinical pathological features and treatments of 67 patients suffering from ENEC who underwent surgical resection was performed to explore the potential prognostic features and to provide more valuable clinical data for the treatment of ENEC.

## 2 Methods

This study collected data from 4135 patients who underwent radical resection of EC at Jiangsu Provincial People's Hospital and Jiangsu Provincial Tumor Hospital from January 2008 to December 2014. Among them, 92 patients were diagnosed with ENEC on pathology and immunohistochemistry (IHC), accounting for 2.2% of the patients undergoing surgical resection for EC during the same period, but 25 lacked follow-up information. The clinical data included the following: patient's age, sex, tumor site, postoperative pathology (postoperative pathology this is defined as pathological cytology analysis of the surgical resections of lesions made into specimens, pathological cytology under the microscope, the results of examination microscopy, and is the absolute standard of diagnosis), adjuvant therapy, survival status, and survival time. The pathological features included the type of ENEC, the degree of differentiation, the tumor size, the tumor–node-metastasis (TNM) stage, and the presence of lymphatic infiltration. Important immune indicators included the following: chromogranin A (CgA), synaptophysin, and the Ki-67 index. Tissue specimens from 67 cases of ENEC were routinely processed and then embedded in paraffin. After sectioning, they were stained using hematoxylin eosin and evaluated. All specimens were immunohistochemically examined using the EnVision 2-step method. The location of the tumor within the esophagus was established based on endoscopic results and divided into the following 3 sections: neck/upper (15–25 cm from the incisors), middle (25–30 cm from the incisors), and lower (30–40 cm from the incisors) esophagus. ENEC staging was determined based on the 2017 American Joint Commission on Cancer (AJCC) TNM staging system (8th edition) for esophageal squamous cell carcinoma.^[7]^ All patients were followed up by telephone. The final follow-up date was set as February 5, 2018. The time of the survival was determined starting from the day of esophagectomy to the date of death or the final follow-up. All of the patients provided informed consent before participating in the study. SPSS 21.0 (SPSS Corp, Chicago, IL) software was used for statistical analysis, and Graphpad software (USA) was used to plot survival curves. Categorical data are reported as the means ± standard deviation, and numerical data were evaluated with a chi-squared test. A Kaplan–Meier survival analysis was performed. Cox regression models were adopted to assess prognostic factors. A 2-tailed *P*-value that was <.05 was considered to be statistically significant.

## 3 Results

### 3.1 Patient characteristics

**Figure 1:**
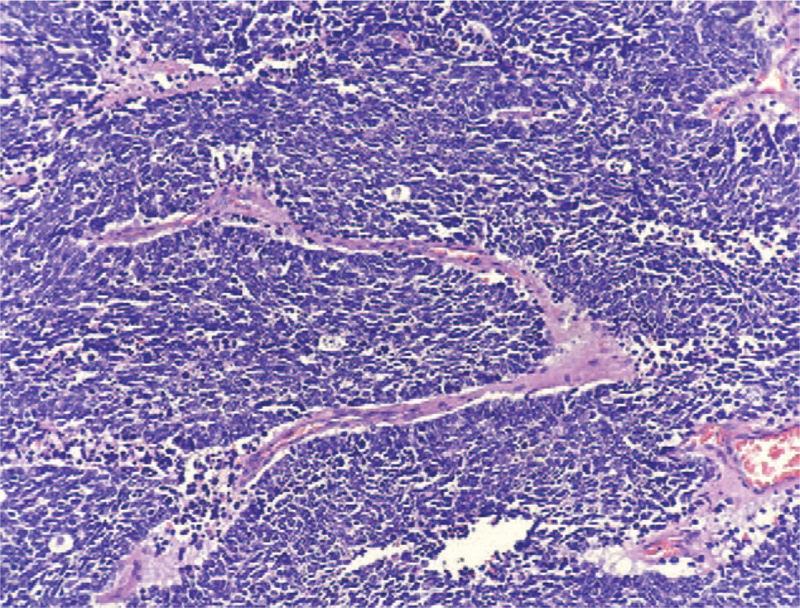
Morphology of small cell carcinoma ×100.

**Figure 2:**
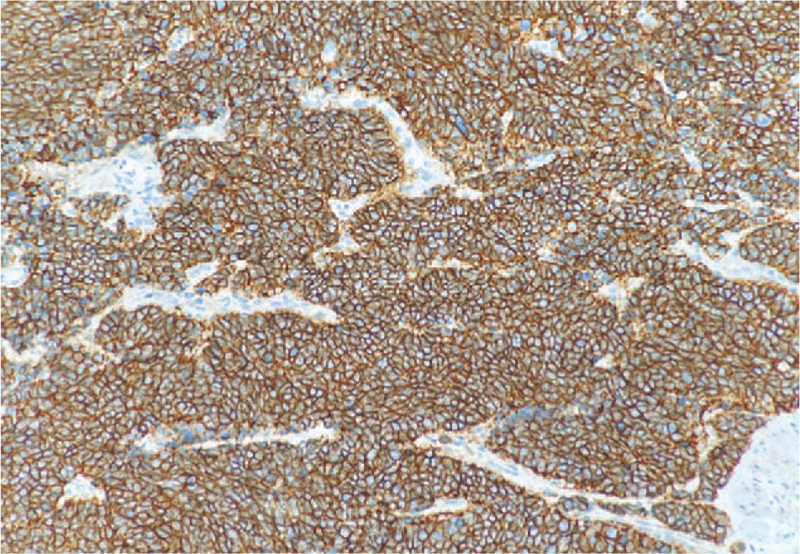
Syn was diffusely expressed ×100.

**Figure 3:**
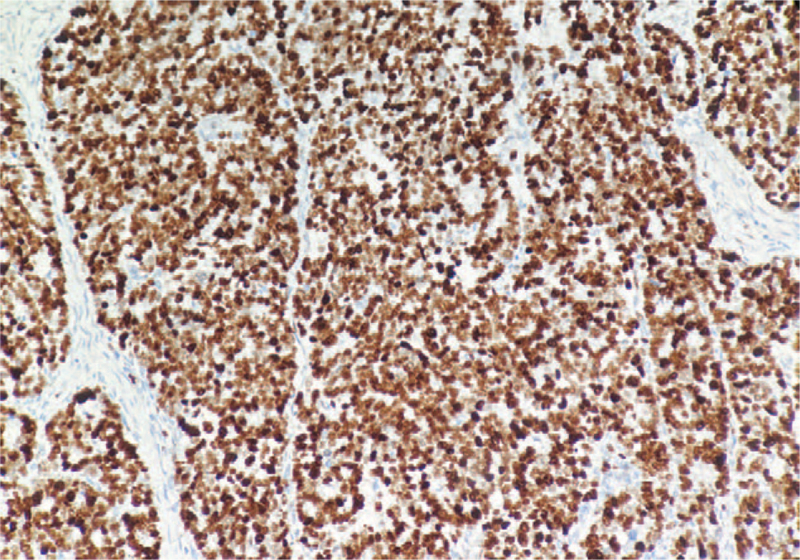
Ki-67 expression ×100.

**Figure 4:**
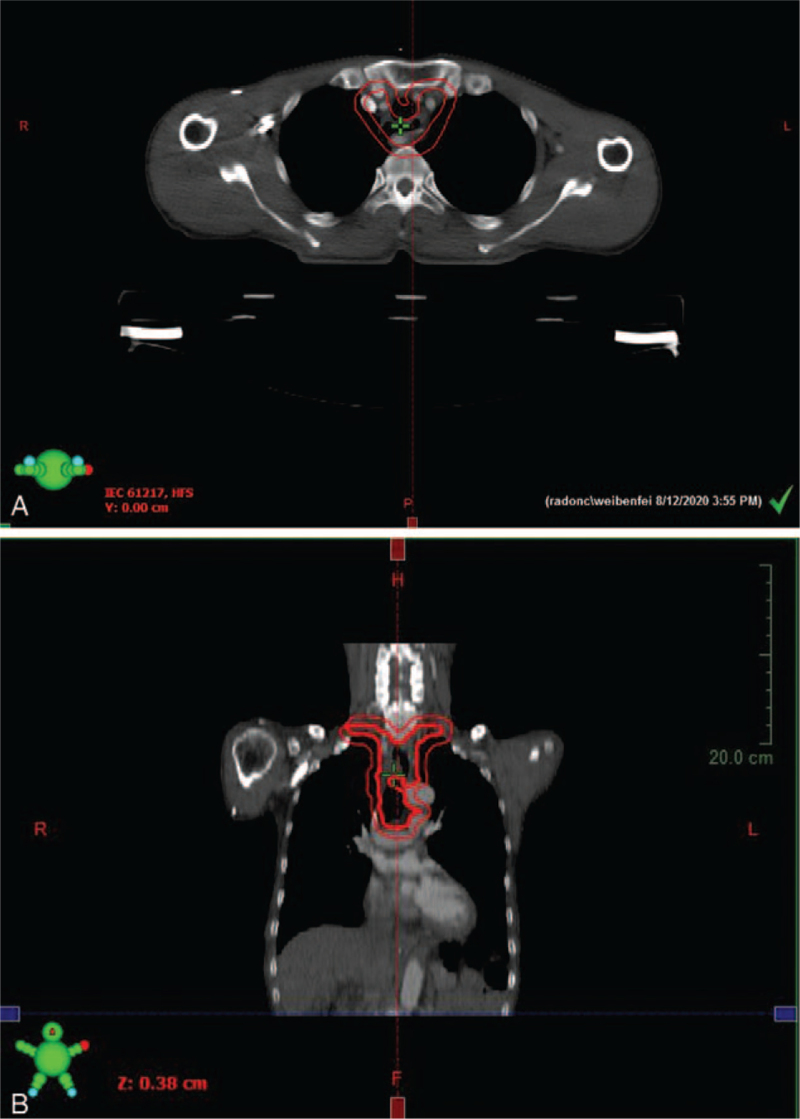
Target delineation of postoperative adjuvant radiotherapy (A: Transverse position, B: sagittal position). The inner red line is CTV (clinical target volume) and the outer red line is PTV (planning target volume).

The preoperative clinical diagnosis of all patients was resectable ENEC (without distant metastasis). Table 1 gives a summary of the clinical features for the 67 patients. The mean age of all patients was 63.69 ± 7.15 years (range 41–79 years), and the ratio of males to females was 5.7:1. More than half of the ENEC was located in the lower esophagus (59.70%), and the postoperative pathology of 40 patients (59.70%) revealed lymph node metastasis. Nine patients had vascular tumor emboli (13.43%), and 4 patients (5.97%) had nerve involvement. All patients with ENEC had detailed pathological findings, IHC results, and IHC maps. Pathologically, the majority of patients had ulcerative type (59.70%), 10 patients (14.93%) had umbrella type, and the other 17 (25.37%) cases were plaque type, protruding type, and superficial phenotypes. In terms of the pathological type, there were 25 cases (37.31%) of large cell NEC, 26 cases (38.81%) of hybrid NEC, and 16 cases (23.88%) of small cell NEC (Fig. 1). The IHC analysis revealed the following: synaptosomes (Syn)/CgA staining +/+ in 53.73% of cases, Syn/CgA staining +/− in 41.79% of cases (Fig. 2), and Syn/CgA staining −/+ in 4.48% of cases. In all cases, the Ki-67 index was greater than 20% (Fig. 3). Postoperative pathological staging revealed 9 patients who had stage I disease, 23 patients who had stage II disease, 31 patients who had stage III disease, and 4 patients who had stage IV disease. In general, 43 patients (64.18%) underwent a postoperative adjuvant treatment (chemotherapy and/or radiation therapy) (Fig. 4A and B), while 24 patients (35.82%) did not. Table 1 shows that the *P*-values in each group were >.05, indicating that the characteristics of the 2 groups of patients who underwent a postoperative adjuvant therapy and those who did not was balanced, with no significant differences.

**Table 1:** Clinical characteristics of patients with esophageal neuroendocrine carcinoma in our study (N = 67).

	Postoperative adjuvant therapy		Total		
Variable	Yes = 43N (%)	No = 24N (%)	n = 67N (%)	Chi-square (χ^2^)	*P*-value
Sex					
Male	39 (58.21)	18 (26.87)	57 (85.07)	2.989	.149
Female	4 (5.97)	6 (8.96)	10 (14.93)		
Age					
<60	13 (19.40)	5 (7.46)	18 (26.87)	0.693	.567
≥60	30 (44.78)	19 (28.36)	49 (73.13)		
Size					
<2 cm	8 (11.94)	4 (5.97)	12 (17.91)	0.039	1.0
≥2 cm	35 (52.24)	20 (74.07)	55 (82.09)		
Pathological type					
Ulcer type	27 (40.30)	13 (19.40)	40 (59.70)	1.254	.788
Medullary type	5 (7.46)	5 (7.46)	10 (14.93)		
Uplift type	4 (5.97)	3 (4.48)	7 (10.48)		
Other	7 (10.48)	3 (4.48)	10 (14.93)		
Histopathology					
Small cell	11 (16.42)	5 (7.46)	16 (23.88)	0.786	.686
Large cell	17 (25.37)	8 (11.94)	25 (37.31)		
Mixed	15 (22.39)	11 (16.42)	26 (38.80)		
Primary site					
Upper/middle	16 (23.88)	11 (16.42)	27 (40.30)	0.476	.605
Lower	27 (40.30)	13 (19.40)	40 (59.70)		
Vascular cancer thrombi					
Yes	6 (8.96)	3 (4.48)	9 (13.43)	0.028	1.0
No	37 (55.22)	21 (31.34)	58 (86.57)		
Nerve invasion					
Yes	2 (2.99)	2 (2.99)	4 (5.97)	0.372	.614
No	41 (61.19)	22 (32.84)	63 (94.03)		
Number of lymph nodes					
<12	14 (20.90)	10 (14.93	24 (35.82)	0.556	.596
≥12	29 (43.28)	14 (20.90)	43 (64.18)		
Syn/CgA staining results					
+/+	25 (37.31)	11 (16.42)	36 (53.73)	1.904	.431
+/−	17 (25.37)	11 (16.42)	28 (41.79)		
−/+	1 (1.49)	2 (2.99)	3 (4.48)		
Differentiation degree high/ medium	4 (5.97)	3 (4.48)	7 (10.48)	0.168	.695
Low	39 (58.21)	21 (31.34)	60 (89.55)		
Histological stage					
I	4 (5.97)	5 (7.46)	9 (13.43)	1.843	.407
II	15 (22.39)	8 (11.94)	23 (34.33)		
III/IV	24 (35.82)	11 (16.42)	35 (52.24)		

### 3.2 Treatment and prognosis

**Figure 5:**
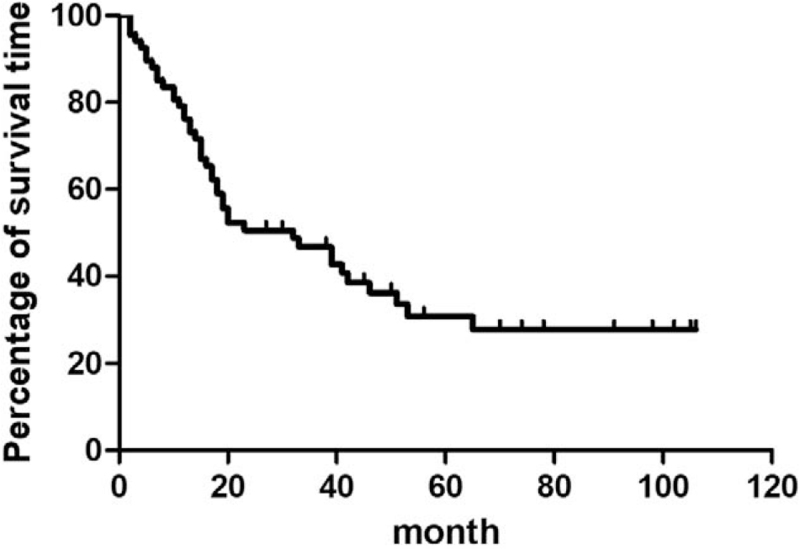
Overall survival (OS) curve of ENEC patients. It showed that survival proportion decreased with the time extending.

**Figure 6:**
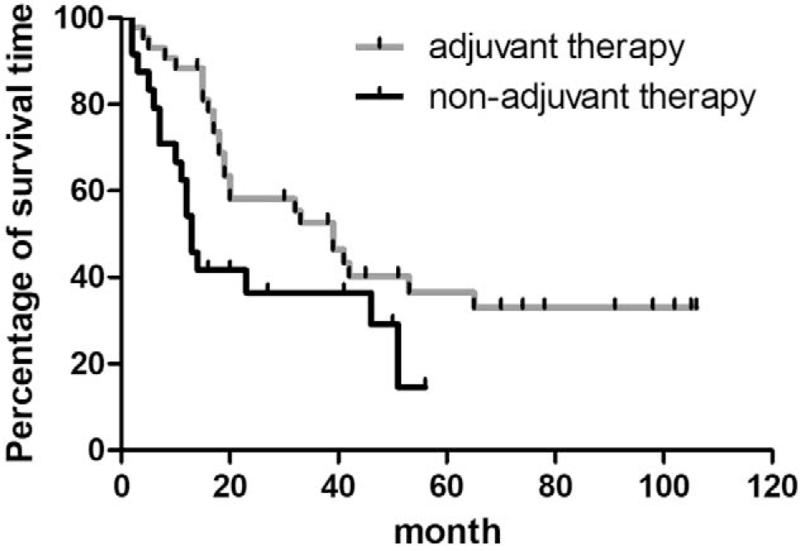
Overall survival curves of adjuvant therapy group vs nonadjuvant therapy. Overall survival in adjuvant group was longer than nonadjuvant treatment group.

All 67 patients underwent radical esophagectomy associated with regional lymph node dissection, 22 patients underwent left thoracic esophagostomy associated with the 2-field lymph node dissection, and 37 patients underwent right thoracic esophagectomy associated with the 2-field lymph node dissection. Six patients underwent thoracoscopic esophagectomy combined with 3-field lymph node dissection, whereas 2 patients underwent radical thoracoscopic esophagectomy combined with 3-field lymph node dissection. R0 resection was performed in 66 patients, and R1 resection was performed in only 1 patient (postoperative pathology suggested positive anastomotic margins). Ninety-two patients were included and followed up, of whom 43 patients died and 24 patients survived. Twenty-five patients (27.17%) were lost to follow-up. A univariate Cox proportion hazards model showed that postoperative adjuvant therapy, age, and lymph node metastasis were associated with prognosis (*P* < .05), but gender, tumor location, tumor histological morphology, pathological type, the presence of vascular tumor emboli or nerve invasion, the number of lymph node dissections, the degree of differentiation, the Syn/CgA staining results, and TNM staging were not associated with prognosis. There was no significant difference (*P* > .05) (Table 2). A multivariate Cox proportional hazards model showed that postoperative adjuvant therapy was a significant independent prognostic factor for ENEC, and postoperative adjuvant therapy clearly affected overall survival (OS), with statistical significance (*P* = .022) (Table 3). In the Kaplan–Meier survival analysis, all patients had an OS of 32 months (interquartile range [IQR]: 12.83–511.17 months). The 1-year and 3-year survival rates for the group were 76.11% and 46.80%, respectively (Fig. 5). In this study, patients who underwent adjunctive therapy had an average survival time of 39 months (IQR: 27.068–50.932 months), while those who did not undergo adjuvant therapy had an average survival time of 13 months (IQR: 10.129–15.871 months). The survival time was reasonably longer in the treated group than in the untreated group (hazard ratio = 0.47; 95% confidence interval: 0.23–0.94; *P* = .034) (Fig. 6).

**Table 2:** Univariate cox model with esophageal neuroendocrine carcinoma (N = 67).

Variable	Hazard ratio	95% CI	*P*-value
Postoperative adjuvant therapy			.039
No	1		
Yes	0.515	0.275 to 0.966	
Sex			.521
	Female	1	
	Male	1.358	.533 to 3.461
Age			.017
	<60	1	
	≥60	2.70	1.192 to 6.115
Primary site			
	Cervical/upper	1	.783
	Middle	1.64	.218 to 12.358
	Lower	1.361	.183 to 10.099
Size			.638
	<2 cm	1	
	≥2 cm	1.233	.515 to 2.9501
Pathological type		0.605	
	Ulcer type	1	
	Medullary type	1.245	.538 to 2.88
	Uplift type	0.712	.249 to 2.038
	Other	0.583	.204 to 1.671
Histopathology			0.929
	Pure	1	
	Mixed	0.929	.501 to 1.761
	Small cell carcinoma	0.766	.18 to 3.267
Vascular cancer thrombi			.167
	No	1	
	Yes	1.789	.785 to 4.081
Nerve invasion			.972
	No	1	
	Yes	1.025	.246 to 4.275
Lymph nodes metastasis			.012
	No	1	
	Yes	2.344	1.209 to 4.545
Number of lymph nodes			.244
		1	
	≥12	1.471	.768 to 2.816
Syn/CgA staining results			.929
+/+	1		
+/−	0.939	0.501 to 1.761	
−/+	0.766	0.18 to 3.267	
Grade		0.446	
Grade I/II	1		
Grade III/IV	1.267	0.689 to 2.33	
Histological stage		0.645	
I	1		
II	1.243	0.41 to 3.765	
III	1.394	0.472 to 4.112	
IV	2.341	0.582 to 9.422	

**Table 3:** Multivariate cox model with esophageal neuroendocrine carcinoma.

Variable	Hazard ratio	95% CI	*P*-value
Vascular cancer thrombi			
Yes	1		.160
No	1.828	0.789 to 4.240	
Lymph nodes metastasis			
Yes	1		.237
No	0.671	0.347 to 1.299	
Postoperative adjuvant therapy			
Yes	1		.022
No	0.475	0.252 to 0.897	

## 4 Discussion

ENEC is an uncommon malignancy of the digestive system. A US study included 42 cases of ENEC diagnosed over 20 years, accounting for 1.26% of esophageal malignancies and 1% of the gastrointestinal neurosecretory tumors.^[8]^ In this study, the occurrence of EC was 2.2%, similar to that reported in domestic and international literature. Huang et al^[9]^ investigated ENEC from 2004 to 2010 in a cancer pathology database and found that ENEC occurred in the lower esophagus because neuroendocrine cells are mainly in the lower esophageal mucosa.^[10,11]^ In this study, the lesions were mainly located within the middle and lower esophagus in 97% of cases. The clinical manifestations of ENEC are similar to other ECs. Other rare manifestations are related to its secretion of hormones. The clinical manifestations of ENEC include dysphagia, loss of weight, and chest pain.^[10,12]^ Therefore, diagnosing ENEC based on the clinical manifestations is difficult, and pathological and immunological markers are needed to help confirm the diagnosis.

In recent years, with the continuous improvement in diagnostic techniques, doctors have learned about the anatomical, morphological, and immunohistochemical characteristics of different types of NEC. The most common pathological form of the disease is the medullary type, followed by the ulcerative type and then the umbrella type. In this study, the most common pathological morphology of esophageal neoplasms was the ulcerative type in 59.7%, followed by the umbrella type, accounting for 14.93%, which was slightly different from other neuroendocrine neoplasms. The microscopic appearances of these tumors are small cell, large cell, and mixed types. The large cell type is the most common, followed by mixed and small cell types. Deng et al^[13]^ reported that the incidence of mixed ENEC was 22.4% (11/49), and all patients had squamous cell carcinoma, which might be associated with the high occurrence of squamous cell carcinoma in China. In this study, small cell carcinoma was responsible for 23.88% of all types, large cell ENEC accounted for 37.31%, and mixed ENEC accounted for 38.8%. IHC is an important molecular biological method for diagnosing ENEC. The immunophenotype of ENEC has both neuroendocrine and epithelial properties. Positivity for neuroendocrine markers is higher than that for epithelial markers.^[12,14]^ The World Health Organization recommends synaptophysin and CgA as required markers for the diagnosis of neuroendocrine tumors. Synaptophysin was diffusely expressed in tissues. CgA was focally or weakly expressed, and the sensitivity of synaptophysin was higher than that of CgA, but synaptophysin was less specific. Huang et al showed that immunohistochemical staining technology can help to increase the detection rate of NEC,^[13]^ The positive rates of synaptophysin and chromogranin were 90% and 20%, respectively. In this study, IHC revealed the following: Syn/CgA staining +/+ in 53.73% of cases, Syn/CgA staining +/− in 41.79% of cases, and Syn/CgA staining −/+ in 4.48% of cases. Previous studies have reported controversial results regarding whether the level of Syn/CgA expression affects prognosis.^[15]^ The Cox proportional hazards model showed that these markers were not related to prognosis in this study, and the main role of these markers is to assist in the clinical diagnosis.

Currently, there are no guidelines for the treatment of ENEC, possibly due to the lack of a remarkable number of ideal clinical studies for ENEC.^[5,9]^ In recent years, with the increases in incidence rate, some experience has been accumulated, and a comprehensive treatment model for surgical therapy as well as postoperative adjuvant therapy has attracted considerable attention. It is well known that the choice of therapy is based on the clinical stage. For early and mid-stage patients, radical curative resection combined with lymph node dissection is superior to radiotherapy, chemotherapy, and combined radiotherapy and chemotherapy. Maru et al^[16]^ conducted an analysis of 44 cases diagnosed with small cell carcinoma of the esophagus. All of these patients underwent radical esophageal resection and lymphadenectomy. The results showed that radical esophagectomy and lymph node dissection were the primary treatment options for early and mid-stage esophageal small cell carcinoma, especially in patients without regional lymph node metastases. In the preoperative evaluations in this study, all patients had no distant metastasis, and radical resection of the esophagus combined with lymph node dissection was the first choice of treatment. Regional or distant lymph node metastases in patients with ENEC affect prognosis.^[17–19]^ Xie et al^[20]^ showed that postoperative lymph node metastasis and tumor thrombi had an impact on prognosis. A univariate Cox proportional hazards model revealed that lymph node metastasis and vascular tumor emboli were significantly associated with prognosis in this study (*P* < .05).

Patients undergoing radical esophagectomy for ENEC have a high risk of recurrence or metastasis after surgery. Thus, preoperative or postoperative chemotherapy may be the key to facilitating patient survival.^[21]^ However, currently, there is no unified standard of postoperative adjuvant therapy. Ding et al^[22]^ summarized the survival time of 106 patients with limited-stage small cell NEC being treated with different modalities.^[12]^ The 5-year survival rate of patients who underwent surgery or radiotherapy alone was 0%, and the average survival time was 11 months. In contrast, the 5-year survival of patients who underwent surgery combined with radiotherapy or chemotherapy was 27.2%, and the average time of survival was 22 months. Patients who underwent surgery combined with chemotherapy and/or radiotherapy had a longer survival time than those who underwent surgery alone (*P* = .001). The univariate and multivariate analysis revealed that chemotherapy was an independent prognostic factor. Kim et al^[23]^ evaluated 40 patients with limited-stage small cell NEC and found that those who underwent radical surgery as well as postoperative chemotherapy had a better survival advantage. The most commonly used chemotherapy regimen is platinum-based combination therapy with 2 drugs.^[4]^ At present, there are no uniform radiotherapy standards for ENEC. Generally, based on the principles of radiotherapy for EC, appropriate radiotherapy doses are used. The target area includes the tumor and enlarged regional lymph nodes.^[24,25]^ Postoperative NEC patients were included in this study, and they were treated with radiotherapy and/or chemotherapy. The average time of survival was 32 months (IQR: 12.83–51.17 months), which was significantly shorter than the 54.8-month OS of patients with esophageal squamous cell carcinoma,^[26,27]^ illustrating the poor prognosis of ENEC. The multivariate Cox proportional hazards model showed that postoperative adjuvant therapy was an independent prognostic factor for ENEC. The average time of survival was 39 months (IQR: 27.068–50.932 months) in patients who received adjuvant therapy and 13 months (IQR: 10.129–15.871) in those who did not receive adjuvant therapy. Patients treated with postoperative adjuvant therapy had significantly prolonged survival times (*P* < .05) compared with untreated patients (hazard ratio = 0.47; 95% confidence interval: 0.23–0.94; *P* = .034).

This study also has several limitations that should be noted. Due to clinical practice needs, ENEC staging is determined according to the UICC-AJCC TNM, 7th edition staging system for EC.^[2,28]^ This study used the 8th edition of the UICC-AJCC staging system for EC from 2017; however, the univariate and multivariate analysis did not show that staging was associated with prognosis, and there were conflicting findings with another study,^[28]^ perhaps due to the small sample size. Furthermore, there may be bias because of the retrospective nature of the analysis or heterogeneity of the population. Moreover, in the later follow-up, the rate of loss to follow-up was noted to be very high (27.17%), inevitably affecting the results of this study.

## 5 Conclusion

ENEC is a rare invasive gastrointestinal malignancy with a poor prognosis. This study retrospectively analyzed 67 patients who underwent surgical resection. Through telephone follow-up, survival analysis of the 67 patients was conducted using a univariate Cox regression model. The results revealed that lymph node metastasis and vascular tumor embolism were associated with a poor prognosis, whereas postoperative adjuvant therapy with a good prognosis; however, the multivariate analysis revealed that the postoperative adjuvant therapy was an independent prognostic feature of ENEC. Postoperative adjuvant treatment significantly prolonged patient survival. Postoperative adjuvant treatment methods still require additional prospective studies in the future.

## Author contributions

**Conceptualization:** Shenxiang Liu.

**Formal analysis:** Xiaolin Ge.

**Funding acquisition:** Xiaolin Ge.

**Investigation:** Zhenzhen Gao.

**Methodology:** Zhenzhen Gao.

**Project administration:** Qing Zhou.

**Resources:** Qing Zhou.

**Software:** Yu Shi.

**Supervision:** Yu Shi.

**Validation:** Wangrong Jiang.

**Visualization:** Min Yang.

**Writing – review & editing:** Xinchen Sun.
